# Evaluating reliability and risk of bias of *in vivo* animal data for risk assessment of chemicals – Exploring the use of the SciRAP tool in a systematic review context

**DOI:** 10.1016/j.envint.2020.106103

**Published:** 2020-10-22

**Authors:** Jennifer Waspe, Thuy Bui, Laura Dishaw, Andrew Kraft, April Luke, Anna Beronius

**Affiliations:** a Institute of Environmental Medicine, Karolinska Institutet, Sweden; b Department of Environmental Science and Analytical Chemistry, Stockholm University, Sweden; c United States Environmental Protection Agency, USA

**Keywords:** SciRAP, IRIS, OHAT, ToxRTool, Risk of bias, Reliability, Systematic review

## Abstract

Within the field of health risk assessment, it is essential that evaluations of reliability or validity of toxicity data are conducted with structure and transparency. To this end, different tools for evaluating toxicity studies have been developed by different groups and organizations, for different specific purposes. The Science in Risk Assessment and Policy (SciRAP) tool was developed for use in the regulatory health risk assessment of chemicals and to promote structured and transparent evaluation of study reliability within European regulatory frameworks. As such, the SciRAP tool is not specifically tailored for use in a systematic review context. However, in light of the current movement towards applying systematic review in the field of environmental health and chemical assessments and European chemicals regulation, we were interested in exploring how SciRAP could be applied in such a context. To achieve this, the scope of the SciRAP tool was first compared to two tools developed based on systematic review principles at the US Environmental Protection Agency’s IRIS program and the National Toxicology Program’s Office of Health Assessment and Translation (OHAT). Next, the SciRAP and IRIS tools were both applied in a case study to evaluate the same nine *in vivo* animal studies and the resulting evaluations were compared.

The SciRAP tool was found to address the majority of the elements included for study evaluation in the OHAT and IRIS tools. In the case study, no major differences were found in the conclusions drawn when using SciRAP or IRIS tools. However, future developments to bring the SciRAP tool more in line with systematic review principles were identified and are discussed.

Overall, this work illustrates the advantages of applying structured and pre-defined methods for study evaluation and provides a unique case study comparing the impact of using different tools for evaluating animal toxicity studies.

## 1. Introduction

An integral component of regulatory health risk assessment is to determine the extent to which relevant studies can be relied upon to inform conclusions regarding the risks of chemical exposure. Consequently, it is essential that evaluations of study quality are conducted in a systematic and transparent manner that also promotes consistency between evaluators, i.e. that all relevant study aspects are considered during evaluation and that all evaluators consider the same study aspects. To this end, methods and tools for study evaluation that include predefined evaluation questions, considerations or criteria can be very helpful. Although it has been recommended that structured and transparent methods for evaluating data quality should be incorporated into hazard and risk assessments (EFSA 2015; 2017; NAS 2011; 2014; SCHEER 2018; Wikoff et al. 2018), there is currently no agreed upon and harmonized approach promoted for chemical assessments (Lynch et al. 2016).

Developing a standardized approach to study evaluation within the field of chemical risk assessment poses a number of challenges. These include producing a tool that promotes consistency between evaluators whilst remaining flexible enough to be applicable to different types of studies and endpoints, as well as accommodating how the requirements of an evaluation may change with time or context. An additional consideration is the intended purpose of the risk assessment and the varying specifications of different regulatory frameworks. Furthermore, the terminology used to describe study quality varies across different contexts, areas and settings. Internal validity or reliability are examples of terms commonly used in different contexts; these and other definitions relating to study quality are further defined in Text Box 1.

Systematic review methodology, which has established use for study evaluation and evidence integration in medicine and epidemiology, is being increasingly adopted into the area of regulatory hazard and risk assessment of chemicals (Whaley et al. 2016; Ågerstrand and Beronius 2016). For example, implementation of systematic review practices is mentioned in the newly adopted European Union (EU) criteria for assessment of endocrine disrupting properties of plant protection products (Regulation EU 2018/605) and biocides and biocidal products (Regulation EU 2017/2100). This presents new challenges to chemical risk assessors, who may be less familiar with the specific rigor required by the systematic review process (particularly consideration of risk of bias) but are increasingly being required to conduct risk assessments in accordance with systematic review principles.

The study evaluation tool developed within the Science in Risk Assessment and Policy (SciRAP) initiative is an example of a tool developed in the context of regulatory health risk assessment. It is intended as a pragmatic aid for risk assessors and founded on requirements for study evaluation which are familiar to this field (e.g. principles for evaluating “reliability” and considerations of criteria in standardized toxicity test guidelines). As such, one specific aim of the work presented here was to explore differences and similarities between evaluating studies for use in risk assessment, using the SciRAP tool, and using tools designed for evidence appraisal in a systematic review scheme.

First, we examine the structure of several different study evaluation tools, comparing the scope of stand-alone criteria-based tools developed for evidence appraisal in regulatory health risk assessment (ToxRTool and SciRAP), and tools developed as part of a broader systematic review scheme (OHAT RoB tool and the US EPA IRIS tool). We also present our experiences evaluating a small sample of *in vivo* animal toxicity studies, in this case comparing evaluations conducted using SciRAP to those using the IRIS tool. The purpose of this case study was to illustrate, with examples, differences in study evaluations that may occur using these tools.

Importantly, the work presented here is not intended as validation of the use of the SciRAP tool in a systematic review context. However, this work does lend itself to consideration of how SciRAP could be developed in the future to be more in line with systematic review methodology as its application is being increasingly implemented in regulatory health risk assessment, and this is also discussed.

## 2. Tools for evaluation of animal toxicity studies in the regulatory context

Over the past 20 years, several tools have been designed and applied in the field of health risk assessment, to aid the process of improving structure and transparency in the evaluation of animal toxicity studies.

The Klimisch categorization method (Klimisch et al., 1997) was the established method for evaluation of study quality for regulatory hazard and risk assessment of chemicals, especially for chemicals regulation within the European Union (EU). The approach introduced by Klimisch and co-workers in 1997 assigned different categories of reliability to empirical animal toxicity data as follows: 1) “reliable without restrictions”, 2) “reliable with restrictions”, 3) “not reliable” and 4) “not assignable”). Reliability, in this context, encompasses aspects of both reporting quality and methodological quality, referring to the consistency and reproducibility of study findings (see Text Box 1). Reliability is the term commonly used in the setting of regulatory hazard and risk assessment within EU chemicals regulation. Although Klimisch et al. listed considerations and prompts to be used by the reviewer to categorize the data, it lacked a systematic approach and guidance needed to ensure consistency between evaluators and across evaluations (Kase et al. 2016). In the Klimisch method, study evaluation is dependent on the adequacy of the reporting of study design, conduct and results and whether it adheres to standardized test guidelines (e.g., OECD test guidelines) and good laboratory practices (GLP). Since the Klimisch method was developed, it has been widely debated whether studies should be considered of lower quality, by default, simply because of a lack of adherence to standardized test guidelines and GLP (Hale and Griffiths 2015; Samuel et al. 2016).

### 2.1. ToxRTool

In 2009, the Toxicological data Reliability Assessment Tool (ToxRTool) was introduced to facilitate study quality evaluation of *in vitro* and *in vivo* studies according to the Klimisch categories, particularly to aid distinction between categories 2 and 3 (defined above). (Schneider et al. 2009). Specifically, the aim of ToxRTool was to improve harmonisation and transparency in reliability assessments in accordance with REACH legislation, by introducing a standardized set of criteria to be addressed and scored (Schneider et al. 2009). The original context for developing the ToxRTool was to support establishing evidence-based practice in toxicology, which was a recently adopted concept in the field. ToxRTool was the first proposed method for evaluating toxicological data quality using pre-defined criteria (Schneider et al. 2009). It follows that the tool significantly predates discussions about systematic review methodology and, as such, the potential use of the tool in this setting was not discussed by Schneider et al.

The ToxRTool is a Microsoft Excel-based, binary scoring system, comprised of a checklist of 21 criteria organized across five study areas for *in vivo* studies: 1) Test substance identification, 2) Test organism characterization, 3) Study design description, 4) Study results documentation, and 5) Plausibility of study design and results. The ToxRTool criteria have been listed in Supplemental table 1. The criteria are mainly focused on the reporting of different study aspects. The algorithm gives a higher weighting to certain ‘red’ (key) criteria resulting in a numerical score based upon the assessor’s appraisal of how the criteria are met by a given study. The score is used to assign the appropriate Klimisch category to the study based upon predefined scoring thresholds. However, the application of numerical scores to define reliability has been deemed problematic because these scores do not indicate the study’s particular strengths and weaknesses (Beronius et al. 2018; Weed 2005). Since some aspects of study design and conduct may have a greater impact on the final conclusion about the study reliability than others, identification of specific strengths and weaknesses may be important when concluding on how a study can be used as evidence in hazard and risk assessment. The ToxRTool does not suggest a minimum number of reviewers or a pre-defined approach for resolving discrepancies between evaluators for a given study evaluation.

### 2.2. The SciRAP tool

The Science in Risk Assessment and Policy (SciRAP) approach for evaluation of animal studies was developed in 2014 with the goal of further promoting transparency and structure in study evaluations for regulatory hazard and risk assessment (Molander et al. 2015). An important aim was to develop a pragmatic simple fit-for-purpose tool for study evaluation within the EU regulatory context, however the intention of the SciRAP tool is to be flexible enough to be used to increase structure for study evaluation in other contexts. Importantly, the SciRAP tool was not developed as part of a specific weight of evidence evaluation or systematic review scheme. Like ToxRTool, SciRAP is a criteria-based tool; comprised of a set of criteria that have to be addressed and evaluated. The SciRAP criteria are based on requirements and recommendations for study design, conduct and reporting outlined in standardized and internationally validated OECD test guidelines for the testing of chemicals for health effects (https://www.oecd-ilibrary.org). However, in contrast to the quantitative ToxRTool method, SciRAP utilizes a qualitative approach to study evaluation (Beronius et al 2018). Importantly, the SciRAP tool does not by default award a higher grade for reliability to studies conducted according to standardized test guidelines or GLP.

The SciRAP tool includes specific detailed criteria for evaluation of study reliability and study relevance and is publicly available online (www.scirap.org). Evaluation of relevance was not included in the exercises and comparisons described in this paper. Relevance of animal studies in a risk assessment context relates to how applicable the study is for answering a specific hazard or risk assessment question. The SciRAP tool includes a separate set of criteria to evaluate the study relevance. This aspect may be comparable to evaluation of “indirectness” and “external validity”, which are subsequent steps in systematic reviews using approaches such as GRADE (Morgan et al. 2018; Schünemann et al. 2013). In other words, while SciRAP includes a function to evaluate relevance on the individual study level, in systematic review such aspects are commonly handled in later steps where evidence is integrated.

The evaluation of overall reliability with the SciRAP tool is divided into evaluation of “reporting quality” and “methodological quality”, separately. For *in vivo* toxicity studies, there are 30 predefined criteria that evaluate reporting quality (Supplemental Table 2) and 18 criteria for evaluating methodological quality (Supplemental Table 3). Criteria are evaluated as “fulfilled”, “partially fulfilled”, “not fulfilled” or “not determined” using a web-based form. Criteria considered to be specifically important for the evaluation being conducted may be given increased weight. Conversely, criteria deemed not relevant to the evaluation may be removed. The evaluation is exported to a Microsoft Excel file, providing a qualitative summary of the evaluation in the form of a colour profile for five different aspects of the study (Beronius et al. 2018): 1) test compound and controls, 2) animal model and housing conditions, 3) dosing and administration, 4) data collection and analysis, and 5) funding sources and competing interests. The colour profile thus provides an overview of where the strengths and weaknesses of the study may lie. The SciRAP tool also calculates numerical scores for reporting quality and methodological quality based on the percentages of fulfilled and partially fulfilled criteria, taking any increased weighting of criteria into consideration. Criteria that have been removed do not contribute to the score. There is no defined approach for how the colour profile or score should be used, e.g. to categorize studies into different levels of reliability, and the score is not intended to be used separately to draw conclusions about study reliability but should always be considered together with the colour profile.

Study evaluation using the SciRAP tool is typically conducted for a selected endpoint investigated in the study, which is decided by the hazard or risk assessment question that provides the basis for the evaluation. SciRAP does not dictate a minimum number of reviewers or suggest a standardized means for resolving discrepancies between multiple reviewers. These aspects have to be decided on case-by-case basis, dependent on the purpose of the evaluation being conducted.

## 3. Establishing systematic review methodology in health risk assessment

In the field of healthcare, Cochrane has held a pivotal role in advancing systematic review methodology for the past 25 years (Chandler and Hopewell 2013). Owing to their methodological stringency, breadth of expert involvement and rigorous planning, Cochrane Reviews are generally heralded as the gold standard approach to evidence appraisal in clinical practice (Useem et al. 2015). Cochrane publishes several types of systematic reviews with different methodological protocols for addressing different clinical questions, for example, concerning the effects of clinical interventions, and diagnostic test accuracy. This necessitates evaluation of a range of study types but does not routinely include the *in vivo* animal toxicity studies typical of regulatory health risk assessment. Thus illustrating that the Cochrane approach is not necessarily directly applicable to study evaluation in a risk assessment context. Nevertheless, application of equivalent levels of acumen to the consideration of bias in regulatory health risk assessment is considered an important area of improvement in the health risk assessment field (Whaley et al. 2016).

The approach taken by Cochrane originated as a bias assessment classification system, which was first introduced in 1996 (Chandler and Hopewell 2013) and, over time, evolved into the Cochrane Handbook; the current guidance document for conducting systematic reviews in the context of health intervention (Higgins and Green 2011). Within this handbook, it is acknowledged that a study can be biased despite adherence to standardized testing guidelines, therefore the preferred method for assessing studies is to conduct a risk of bias (RoB) assessment. A RoB assessment is variously described as an evaluation of aspects of quality, reliability or internal validity (Rooney et al. 2016), or the likelihood of a presence or absence of systematic error (Wikoff et al. 2018). Here, emphasis is placed primarily on whether the results of a given study are valid/reliable, as opposed to whether it was conducted according to the highest standards. Thus, a RoB assessment differs somewhat to an assessment of reliability, which was previously the approach used in relation to chemicals regulation. The term RoB is defined in Text Box 1. The Cochrane Handbook also makes the distinction between internal validity and completeness of reporting, arguing that these should be considered separately and not encompassed in a single evaluation of study quality (Higgins and Green 2011).

Discussions pertaining to the advantages of RoB over reliability assessments in conducting high quality systematic reviews are one reason for increasing the integration of systematic review methodology in regulatory health risk assessment of chemicals. Several study evaluation tools for use in this area have been developed specifically to assess various RoB domains. These tools include the National Toxicology Program’s Office for Health Assessment and Translation (OHAT) RoB tool (OHAT 2019); a study evaluation component embedded in the systematic review approach of the US EPA’s IRIS Program (NAS 2018; see https://hawcprd.epa.gov/assessment/497/ for an example of a recent application of this study evaluation approach), the Navigation Guide (Woodruff and Sutton 2014) and SYRCLE (Hooijmans et al 2014). We here refer to these tools as “domain-based” since they are structured based on different issues, or domains, related to RoB that should be addressed during evaluation. Each domain may consist of one or more specific questions.

Similarities and differences between different tools for study evaluation have been investigated previously (Kase et al. 2016, Krauth et al. 2013, Lynch et al. 2016). While there are numerous available tools which could have been included in our comparisons, our aim was primarily to explore the extent to which SciRAP would be fit-for-purpose for conducting study evaluation for chemical risk assessments in a field transitioning increasingly towards systematic review. As such, it was deemed sufficient to do a comparison with the IRIS and OHAT tools, as they have been developed for RoB assessment within broader systematic review schemes. Together, the IRIS and OHAT tools were considered to adequately represent evidence appraisal tools in the context of systematic review. For the purpose of identifying aspects of RoB assessment not covered in SciRAP it was considered unlikely that including additional tools for comparison would reveal further differences.

### 3.1. The IRIS tool

The Integrated Risk Information System (IRIS) program was developed in 1985 as a means to improve consistency in risk assessments undertaken by the US Environmental Protection Agency (US EPA) concerning human exposure to environmental contaminants. Human health hazard and dose–response assessments developed by IRIS have since been used to inform regulatory standards and actions relating to chemical exposure at local and national levels. In response to recommendations from the National Research Council, gradual evolution of the IRIS program brought about a number of improvements to the assessment development process as a whole, and since 2011 the program has been specifically developed to be more in line with systematic review principles (NAS. 2014). The IRIS tool for evaluating *in vivo* animal studies is now part of a broader systematic review approach for developing hazard identification conclusions regarding the potential for a causal relationship between chemical exposure and a specific health effect. Specifically, the study evaluation ratings are used to inform judgements regarding causality during the synthesis and integration of available evidence and in the selection of datasets for dose–response analysis.

The domains within the IRIS tool fall under three evaluation categories: reporting quality; RoB; and study sensitivity (Table 1). Evaluation of reporting quality examines impact of the completeness of the information provided on the study design and results. This category can be used to determine whether studies provide sufficient detail to move forward with the full study evaluation, flag critical missing information, and identify whether author queries for additional details may be necessary early in the review process. It should be noted that the intent of this domain is not to exclude studies from further analysis, although this may be the case if the inadequacy of reporting is egregious. The RoB domains are similar to other RoB-based approaches (Krauth et al. 2013; Lynch et al. 2016; Page et al. 2018) and evaluate potential biases related to study selection, performance, confounding, selective reporting, and attrition. The study sensitivity domains address aspects of the study design and conduct related to the test compound, chemical exposure (timing, frequency, and duration of exposure), outcome evaluation, and results presentation that may influence the ability of the study to detect an effect. Domains are rated as “good”, “adequate”, “deficient”, “critically deficient” or “not reported”. Depending on the domain, these ratings may be made at the level of the study as a whole (e.g., reporting quality), the experiment (e.g., allocation procedures), or each outcome being assessed (e.g., endpoint evaluation methods), such that a study may have multiple ratings for a given domain. The level of granularity for each domain rating is described in the tool. Typically, for a particular study and health outcome the individual domain ratings inform the overall confidence rating (i.e., high, medium, or low confidence, or uninformative, as determined based on the independent reviewers’ expert judgment) thus, an individual study may have confidence ratings that differ across different outcomes.

The IRIS tool may be used in a standalone manner and is operationalized within a Health Assessment Workspace Collaborative (HAWC) platform maintained by US EPA (https://hawcprd.epa.gov/portal/). HAWC provides a publicly available, web-based system for organizing, conducting, and documenting hazard identification and dose–response decisions. In order to reduce subjectivity of the IRIS approach, each study is evaluated by at least two independent reviewers with appropriate expertise and discrepancies between reviewers are resolved (potentially using a third reviewer) prior to assigning a finalized rating to the study/outcome pair. Finalized ratings can be used to generate interactive graphical displays, demonstrating the results collated within HAWC from a group of studies addressing the same outcome.

### 3.2. The OHAT tool

The role of the Office of Health Assessment and Translation (OHAT) is to conduct hazard identification of human exposure to a given environmental substance and provide the public and regulatory bodies with information communicating the level of concern attributable to the substance in question (OHAT 2015). The OHAT RoB tool was developed in 2014, with the intention of increasing objectivity and transparency in relation to evidence synthesis for environmental health. The aim of the tool was to extend the systematic review framework for evaluating clinical studies and adapt it to encompass *in vivo*, *in vitro* and in silico datasets (OHAT 2015). The OHAT RoB tool for evaluating *in vivo* animal studies is incorporated into a seven stage process, referred to as the OHAT Approach to systematic review and evidence integration (for assessing environmental health risks) (Rooney et al. 2014; OHAT 2019), which generates hazard identification risk of bias conclusions. The six RoB domains (selection, performance, attrition, detection, selective reporting and ‘other’) covered within the scope of the OHAT approach are based on guidance from the Agency for Healthcare Research and Quality (AHRQ) as well as the Cochrane Collaboration and others operating within the healthcare remit. Across the domains, there are a total of eleven different questions to address the OHAT RoB domains (Supplemental table 4). An outcome of interest for a given study should be assessed separately by two reviewers. The reviewers judge the risk of bias in each domain to be “definitely low”, “probably low”, “probably high” or “definitely high” according to pre-specified guidance. Any discrepancies between reviewers’ evaluations are resolved through discussion between the reviewers. If discrepancies remain, they will be addressed by the project lead and/or project team (OHAT 2019). The OHAT RoB tool can be applied in a standalone manner and is operationalized within the HAWC platform (www.hawcproject.org).

## 4. Similarities and differences in the scope of IRIS, OHAT, SciRAP and ToxRTool

In the field of chemical risk assessment, there is no single recommended approach to study evaluation. The heterogeneity of endpoints and study types encompassed in this field of study, as well as the varying requirements of individual risk assessments means that establishment of a gold standard method may not be an achievable, or even a desirable, goal. When evaluating toxicological studies for chemical risk assessment, the choice of study evaluation tool will depend on the context of the tool’s application, including the aim of the assessment and problem formulation. In this paper, the evaluation domains as defined within the IRIS tool (Table 1) were used to build a framework against which the other tools were compared. This approach was taken based on the authors’ expertise as developers of the IRIS tool, and therefore greater familiarity with the system as a whole, acknowledging that this introduces an element of bias. However, for this exploratory exercise that bias was not considered critical.

Comparisons across the four tools were made in a tabular format in Microsoft Excel by matching the aspects included in each tool to the nine IRIS domains. Elements covered by ToxRTool, SciRAP and OHAT that were not specifically covered in the IRIS tool were also identified. This format enabled comparisons of the elements covered in the different tools and an illustration of crossover as well as differences across tools. The focus of the comparison was on approaches related to evaluating *in vivo* animal studies and did not include approaches for evaluating *in vitro* or epidemiological studies.

**Fig. 1.**
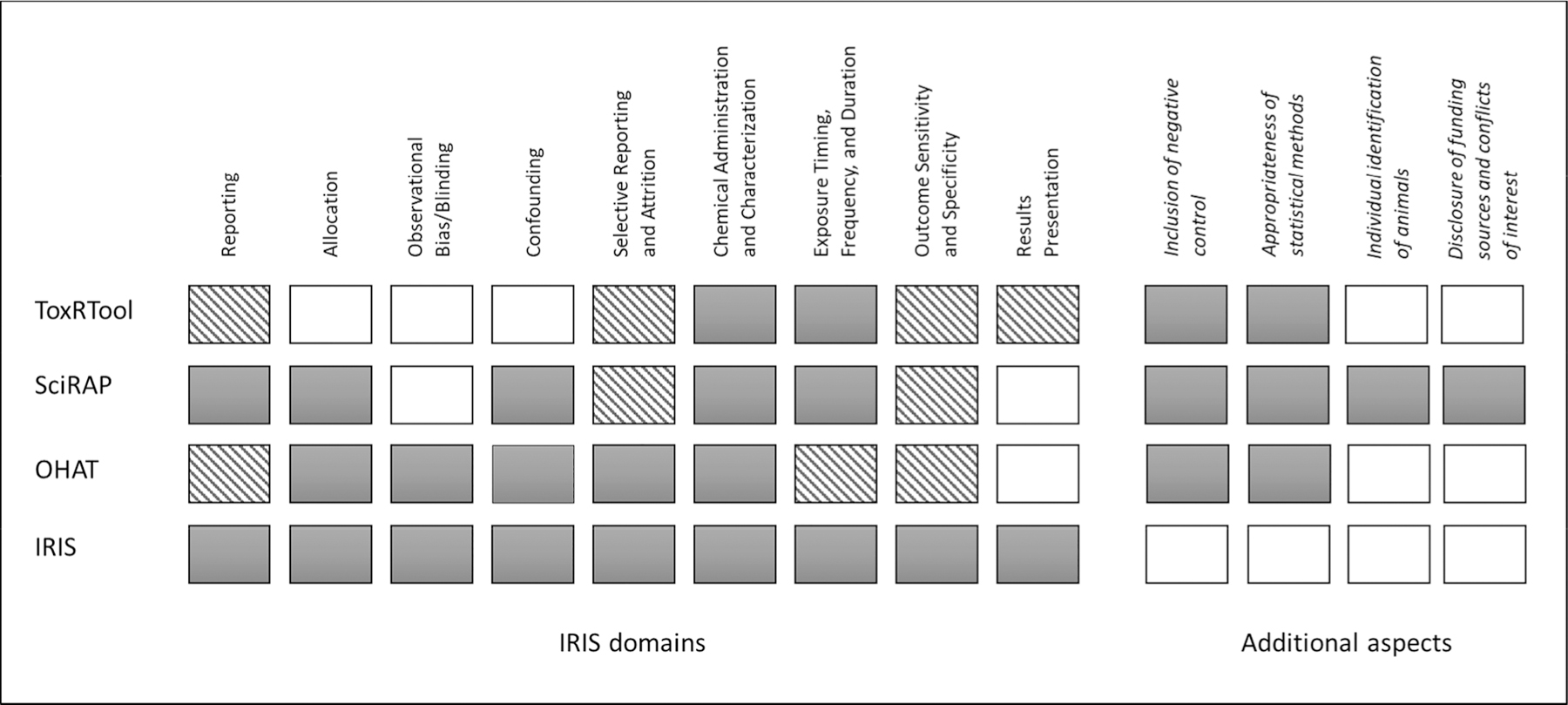
Comparison of the coverage of different elements of study evaluation among the ToxRTool, SciRAP, OHAT and IRIS tools. The nine domains of the IRIS tool were used as the baseline for comparison. In addition, specific additional aspects that were found to be covered by one or more of the other tools but not by the IRIS tool are depicted to the right. Gray blocks = the element is addressed, striped blocks = the element is partially addressed, white blocks = the element is not addressed by the tool. More detail is provided in Table 2.

Comparison of the scope of the different tools and their coverage of the different IRIS domains and prompting questions are summarized in Table 2 and Fig. 1. There was not a complete overlap between the OHAT and IRIS tools, illustrating the different needs and requirements at the organizations that have developed these two domain-based tools.

The OHAT tool was found to fully address five and partially address three of the nine domains described in the IRIS tool. The domain “Results presentation” was found to not be addressed in the OHAT tool. The SciRAP tool was concluded to fully address five and partially address two of the nine IRIS evaluation domains. The domains “Observational bias/blinding” and “Results presentation” were found to not be addressed in the SciRAP tool. Of the tools included for comparison here, the ToxRTool was found to have the least overlap with the IRIS tool, fully addressing two and partially addressing four of the nine domains.

It is important to note that for some criteria, SciRAP and ToxRTool provide additional notes for reviewer guidance regarding aspects a particular criterion might cover, or which could be considered. Examples of this include SciRAP methodological criterion 18 and ToxRTool criterion 20 (see Supplemental Tables 1 and 3). In both of these cases, the additional information for reviewers contains a list of suggested considerations that encompass multiple aspects of study reliability. SciRAP criterion 18 suggests, but does not mandate, consideration of blinding and attrition alongside other prompts such as the measurement of internal dose. Criterion 20 in ToxRTool suggests that in assessing the adequacy of study design, considerations may include: randomised allocation, selective reporting (i.e. inclusion of relevant endpoints) and inadequate statistical evaluation, among others. In these tool comparisons, we decided that considerations listed in this way did not equate to the tool in question definitively addressing a study element or RoB domain. In other words, SciRAP could not be considered to address blinding on the basis of suggestions in the reviewer guidance for criterion 18, as this format does not guarantee (or even require) a reviewer to consider all suggested considerations in every evaluation. Therefore, items listed in these considerations were not taken into account when comparing either tool to the IRIS domains.

Some elements covered by OHAT, SciRAP and ToxRTool were found to not be included in the IRIS tool. These are summarized and discussed as “Additional aspects” in Table 2 and below.

### 4.1. Reporting quality

The IRIS approach for evaluating reporting quality addresses “critical” and “important” information. Critical information is very limited in scope and is considered the minimum amount of information needed for a study to be considered further for study evaluation (i.e., species; test article name; levels and duration of exposure; route; qualitative or quantitative results for at least one outcome of interest). Within the IRIS tool, a study that is missing any piece of critical information is assigned a rating of critically deficient for reporting quality, uninformative for the overall confidence, and is eliminated from further consideration for the systematic review. Important information is much broader in scope and is used to distinguish between “Good”, “Adequate”, and “Deficient” ratings depending on the extent and significance of the reporting omissions. In the IRIS tool, this domain is also used to flag issues where it may be useful for the reviewers to reach out to study authors for clarification that may assist in judging the RoB and study sensitivity domains. For example, if information is missing about the number of animals in each dose group or details about animal husbandry and handling.

Overall reporting quality is not explicitly addressed in the OHAT tool, although certain data extraction elements contribute to the RoB assessment at an earlier stage of the overall OHAT process.

Similar to the IRIS tool, evaluating reporting quality of studies is handled separately in the SciRAP tool. A set of 30 criteria are specifically dedicated to reporting quality and are addressed separately from methodological quality. The SciRAP tool does not specify any reporting criteria that are critical to the evaluation. However, it includes a function that allows the user to increase the weight of criteria they deem as specifically important for study evaluation in the specific case at hand. Similarly, criteria may be removed if considered not relevant in the specific case.

In the ToxRTool, the majority of criteria actually address the level of reporting, albeit in many cases indirectly, with only a few (criteria numbers 13, 16, 19, 20, 21) more directly addressing aspects of methodology or study conduct (Supplemental table 1).

### 4.2. Allocation

The IRIS domain “Allocation” includes the consideration of randomization and that each animal or litter should have an equal chance of being assigned to any experimental group. This domain was found to be covered also by the OHAT and SciRAP tools, but not by ToxRTool.

### 4.3. Observational bias/blinding

The IRIS domain “Observational bias/blinding” includes considerations of concealment of the allocation to different treatments, as well as blinding research personnel to treatment groups when making observations. Neither SciRAP nor ToxRTool were found to address this domain directly. However, in the SciRAP tool the guidance for methodological quality criterion “Other aspects of study design, performance or reporting that influence reliability” includes blinding as an example of additional aspects to consider.

### 4.4. Confounding

This IRIS domain aims to evaluate to what extent experimental conditions were consistent across study/treatment groups. As described in the IRIS tool, this includes considerations of differences between groups with regard to co-exposures, vehicle, diet, palatability, husbandry, health status, etc. SciRAP was found to address this domain via five of its methodological quality criteria. The OHAT tool addresses this domain through consideration of whether experimental conditions were comparable/similar. In addition, consideration of ‘unintended co-exposures’ is listed under the domain ‘other’ in the OHAT tool. In ToxRTool basic information regarding animal husbandry is considered in one of the ToxRTool criteria but no qualitative assessment of experimental conditions is implied. In addition, no ToxRTool criteria are directed towards the identification of co-exposures or use of an appropriate vehicle control.

### 4.5. Selective reporting and attrition

This IRIS domain evaluates two reporting-related forms of RoB: selective reporting and attrition bias. Both components evaluate the potential for bias introduced by authors not providing qualitative or quantitative results for all pre-specified outcomes (i.e., selective reporting) or failing to appropriately account for all tested animals (i.e., attrition bias). The SciRAP tool does not specifically consider whether results for all outcomes indicated as evaluated were presented (either qualitatively or quantitatively) as part of methodological quality of the study. However, SciRAP was concluded to partially address this element through one of its reporting quality criteria (“All results for the investigated endpoints were reported. The most critical results were presented in tables and figures, including description of variation and statistically significant results”, Supplemental Table 2), although this criterion does not directly address bias introduced when reporting is incomplete. This is the only circumstance where a SciRAP reporting criterion was considered to address an IRIS domain other than that of reporting quality.

SciRAP does not include any criteria that directly address attrition. However, consideration of any unexplained animal losses is given as an example in the guidance for the methodological quality criterion “Other aspects of study design, performance or reporting that influence reliability”.

The OHAT tool clearly address both selective reporting and attrition, whereas ToxRTool includes one criterion that addresses selective reporting but no criteria that address attrition.

### 4.6. Chemical administration and characterization

This domain includes considerations related to the purity of the test compound and the accuracy of the dosing. This domain was found to be addressed at comparable level of detail by the OHAT tool, SciRAP tool and ToxRTool.

### 4.7. Exposure Timing, Frequency, and duration

This domain in the IRIS tool focuses on the sensitivity of the exposure regimen. This includes whether the exposure period covered any critical window of sensitivity (if known) relevant to the outcome being evaluated, and whether the duration and frequency of exposure were sensitive for detecting the outcome of interest. The SciRAP tool and ToxRTool were found to include criteria that directly address sensitivity of the study in regard to these aspects. However, it was noted that the OHAT tool focuses the RoB assessment primarily on whether the exposure was applied in a consistent way and not whether a sensitive method was used. Due to the emphasis placed on consistency of the exposure method and time frame, rather than whether these were appropriate for the evaluation of the outcome(s) in question, this was judged to be “partially addressed” by the OHAT tool.

### 4.8. Outcome sensitivity and specificity

This IRIS domain includes considerations of the timing of the outcome assessment, the sensitivity, specificity and validity of the protocol, and the sample size. It was found that none of the other tools include a means for assessing the validity of a given study protocol. The adequacy of sampling in relation to generating reliable results is also considered in the SciRAP tool but not in the OHAT tool or the ToxRTool. It should be noted that the IRIS approach indicates that sample size considerations alone are insufficient to warrant exclusions of studies from further consideration. Consequently, the OHAT, SciRAP and ToxRTool tools were found to only partially cover this domain.

### 4.9. Results presentation

The “Results presentation” domain considers how completely and appropriately the results are presented to allow for an accurate and appropriate interpretation of the study findings, including that results are not presented in a misleading way. In the IRIS approach, this does not encompass an assessment of statistical methodology, which should instead be reserved for statistician input if deemed necessary. In part, this is due to the fact that the IRIS Program workflow includes a dedicated statistics workgroup that can conduct independent statistical analyses if the data are amenable. Therefore, it should be noted that, within the context of the IRIS Program’s workflow, the important aspect of statistical analyses are not overlooked but is more fully evaluated outside the application of the IRIS tool. The OHAT and SciRAP tools were found to not address the element “analysis of results” in accordance with the IRIS tool. However, the ToxRTool incorporates a broader consideration of results credibility as “are the quantitative study results reliable?” and was therefore deemed to partially fulfill this element.

### 4.10. Additional aspects

Some elements to include in the evaluation of *in vivo* animal studies were identified in the SciRAP tool, ToxRTool and OHAT tool that were not covered in the IRIS tool. These are shown in Table 2.

The OHAT tool, SciRAP tool and ToxRTool all address consideration of a concurrent negative control and the appropriateness of the statistical methods used in the study. The OHAT and SciRAP tools include specific elements considering the suitability of the statistical analyses. In addition, the SciRAP tool includes both reporting and methodological quality criteria addressing the means of individual identification of animals. This element was not covered by any of the other tools.

The SciRAP tool also includes criteria for reporting quality that directly address disclosures of funding sources and conflicts of interest (COI). Information on COI can be captured within the data extraction tools for OHAT and IRIS but is not specifically a component of the study evaluation process. COI is not addressed in the ToxRTool. Finally, the OHAT and SciRAP tools include a specific domain or criterion, respectively, for consideration of “other” study elements that can have an impact on study validity or reliability.

## 5. Case study comparing study evaluation using the IRIS and SciRAP tools

To observe how evaluations using tools developed in different contexts compare in practice, a case study was performed using the SciRAP and IRIS tools to evaluate a sample of *in vivo* animal studies. This case study was part of a pilot exercise for the evidence appraisal step of a full systematic review of the flame retardant TPhP. The purpose of including it here was to demonstrate some of the issues which might arise during evaluation of a heterogeneous sample of *in vivo* animal studies, inclusive of different study designs, species and endpoints, which is typical of the literature informing regulatory health risk assessments. Naturally the scope of issues demonstrated is limited by the studies evaluated, and as such this is an example exercise looking at a small sample of studies.

IRIS was selected as the comparison tool owing to the collective expertise of the authors as developers of the IRIS tool and due to the fact that both SciRAP and IRIS incorporate a dedicated reporting quality domain. Since assessment of reporting quality constitutes half of the evaluation process in SciRAP, it was felt that comparison to IRIS would facilitate better examination of the specific aspects of reporting that were similar or different. This is rather to conduct a thorough comparison of the tools than to claim that separate evaluation of reporting quality is an essential component of a RoB assessment. In practice, a judgement on whether reporting is adequate for a study to be included in a systematic review is frequently taken at a different stage to the RoB assessment and it is beyond the scope of this work to discuss the benefits and limitations of each of these approaches.

Although it would have been possible to compare evaluations using IRIS, OHAT and SciRAP, it was felt that a multilevel comparison of numerous evaluations using all three tools would not contribute significantly to the purpose of the study and may compromise clarity.

As the ToxRTool was not designed to address RoB it was not included in this part of the study.

### 5.1. Methods and materials

The case study constituted evaluations of nine *in vivo* animal studies, which were identified during the evidence collection and screening for the systematic review of TPhP (Bui et al., manuscript in preparation). Details about the nine studies included in the case study can be found in Supplemental Table 5. Apart from *in vivo* studies, further inclusion criteria were 1) outcomes relevant to endocrine disruption, 2) vertebrate species and 3) exposure/dosing via feed or injection. Four rat, one hen, two fish and two mouse studies investigating developmental toxicity or neurotoxicity were included. Evaluations were performed by two junior researchers with different academic backgrounds and expertise in medicine and toxicology, or chemistry and environmental science, respectively. Both reviewers had limited previous experience using the SciRAP tool and no previous experience using the IRIS tool. The level of experience of the reviewers was not considered critical to the outcome of the evaluations in this exploratory case study. Neither reviewer received specific training in the use of either tool but instruction on the use of each tool was provided by the tool developers as needed.

The same two reviewers independently read and evaluated each study using both the SciRAP and the IRIS tools. The studies were evaluated in a random order, and the tool used to perform the evaluation first for each study was also decided at random. Despite this randomization procedure, it should be taken into consideration that the repetitive nature of the task and the fact that discrepancies are likely to be sporadic in occurrence, means there is a degree of bias introduced by immediate experience performing the evaluations, which is unavoidable. As questions and concerns arose regarding application of the tools, guidance was provided by the persons responsible for the development of the IRIS and SciRAP approaches. The reviewers were allowed to re-evaluate their responses after receiving clarification. The evaluation results were recorded in a tabular format in Microsoft Excel and compared across both reviewers and tools.

To enable comparisons across the two tools, specific principles for translating the output from the SciRAP evaluations into grades for each IRIS domain were defined by the authors and are shown in Tables 3 and 4. Reporting quality was evaluated using principles outlined in Table 3 and the other five domains were evaluated using principles outlined in Table 4. For each of the nine studies, a rating of “++” (good), “+” (adequate), “-” (deficient) or “- -“ (critically deficient) was applied to each domain. Each study was given overall confidence ratings based on the evaluation with the SciRAP tool and IRIS tool, respectively. The overall rating of “++” (high confidence), “+” (medium confidence), “-“ (low confidence) or “- - “ (uninformative) was determined based on the principles of the IRIS method (Yost et al. 2019), as follows:

- High confidence: No notable concerns are identified (e.g. most or all domains rated Good).
- Medium confidence: Some concerns are identified but expected to have minimal impact on the interpretation of the results. (e.g., most domains rated Adequate or Good; may include studies with Deficient ratings if concerns are not expected to strongly impact the magnitude or direction of the results). Any important concerns should be carried forward to evidence synthesis.
- Low confidence: Identified concerns are expected to significantly impact on the study results or their interpretation (e.g., generally, Deficient ratings for one or more domains). The concerns leading to this confidence judgment must be carried forward to evidence synthesis (see note).
- Uninformative: Serious flaw(s) that make the study results unusable for informing hazard identification (e.g., generally, Critically Deficient rating in any domain; many Deficient ratings). Uninformative studies are not considered further in the synthesis and integration of evidence.

### 5.2. Comparisons of evaluations between tools and reviewers

Outcomes of the SciRAP evaluations from both reviewers across the nine studies are shown in Fig. 2a (reporting quality) and Fig. 2b (methodological quality). In these figures, the reviewers’ evaluations of the SciRAP criteria have been organized according to the structure of the IRIS domains in order to facilitate comparisons between the two tools.

For the evaluation of reporting quality, the SciRAP reporting criteria were divided into those covering “critical information” and “important information”, as described in the tool. The two SciRAP criteria relating to conflict of interests were excluded in Fig. 2a, since they are not covered by the tool.

**Fig. 3.**
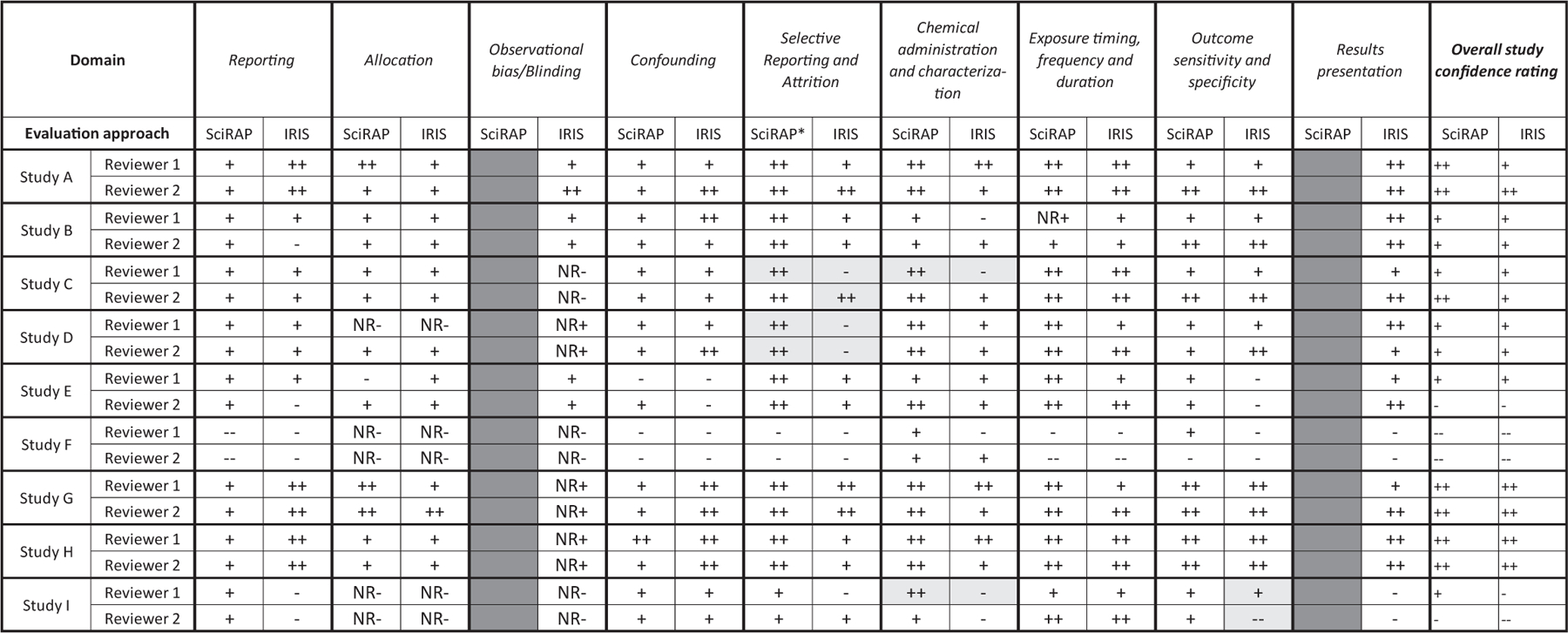
Grading of evaluation domains for the nine studies using the IRIS tools and based on the SciRAP evaluations and principles for grading specified for this case. Dark grey columns depict IRIS subdomains that are not directly addressed by SciRAP criteria. Light grey cells depict instances where the grading differed by more than one level either across tools or between reviewers. “++” = good, “+” = adequate, “−“ = deficient, “−“ = critically deficient, “NR+” = not reported but judged as adequate, “NR-“ = not reported and judged as deficient. *This domain only covered by one SciRAP reporting quality criterion.

The IRIS gradings from both tools are shown in Fig. 3. The two IRIS domains found to not have corresponding SciRAP criteria are marked with dark grey in Fig. 3.

Overall, the evaluations were largely in agreement between the two reviewers for the two tools. There were five instances between tools for the same reviewer and two instances between reviewers using the same tool where a domain grading differed by more than one level (e.g. between “++” and “-“, or between “+” and “- -“). This indicated reviewers had differed significantly in their judgment, i.e. the criterion was evaluated as “fulfilled” by one reviewer but as “not fulfilled” by the other (marked light grey in Fig. 3). Specifically these discrepancies occurred in the following cases: reporting quality criteria 10 (Study A and G), 11 (Study A), 15 (Study B), 24 (Study G and I), 25 (Study C), 26 (Study I) and 27 (Study F), as well as methodological quality criteria 11 (Study F) and 16 (Study D). Whilst it seems acceptable that the domain grading may differ by one level (for example “good” vs “adequate”), the cases where grading varied by more than one level (e.g. “good” vs “deficient”) warrant careful consideration and further discussion. Discrepancies in domain gradings between tools primarily resulted in a lower, more conservative grade with the IRIS tool compared to the SciRAP tool. Notably the discrepancies in individual domains did not result in significant differences between tools or between reviewers in the evaluation of overall study confidence (Fig. 3, right column).

The five instances of discrepancy between tools for the same reviewer occurred in two domains; “Selective reporting and attrition” and “Chemical administration and characterization”. The former was one of three domains found to be only partially covered by the SciRAP tool (due to a lack of criteria for addressing attrition). Consequently, conclusions for this domain were reliant on aspects relating to completeness of reporting alone, and so it follows that SciRAP evaluations had a more favorable grading for this domain than IRIS evaluations. For example, a grading of “++” rather than “-“ was given to this domain for Study C (one reviewer) and Study D (both reviewers) when using SciRAP and IRIS respectively. For study D, both reviewers specifically commented that there was inadequate information in the study to be able to evaluate attrition using the IRIS tool. However, the instructions for IRIS evaluation specifically state that this domain should not be graded as “not reported”, hence the domain was graded as “deficient”. This deficiency in the study was overlooked when using SciRAP and instead the study was rated higher in that regard due to the fact that the reporting was good. Similarly, if there had been serious concerns regarding attrition in the other studies, they are more likely to have been identified using the IRIS tool but could have been overlooked using the SciRAP tool. This provides an example of SciRAP not being sensitive to specific study aspects (attrition), but also highlights the utility of the criteria-based tool in identifying where a study is strong or weak.

In contrast, the second domain where discrepancies between tools were found (“Chemical administration and characterization”) is well covered by several different SciRAP criteria. Two cases of discrepancies occurred in this domain for Reviewer 1. In one instance, the reviewer did not detect potential concerns regarding the administration of test substance when using SciRAP but did note these concerns in the IRIS evaluation. In the second case, the evaluation in SciRAP appears to be less sensitive to a lack of data on purity than the IRIS tool. However, in SciRAP, purity is more directly assessed as part of the reporting quality.

In two instances, the domain-grading differed by more than one grade between the two evaluators. Both instances occurred in the evaluations using the IRIS tool, in the domains “Selective Reporting and Attrition” and “Outcome sensitivity and specificity”. It is likely that these discrepancies were due to differences in how the reviewers interpreted the IRIS prompting questions for these domains and in the case of those studies. Further training and experience with applying the IRIS tool may result in more consistent evaluations between reviewers.

This case study constituted evaluation of a small sample of studies concerning one specific subject area. As such, it can only be considered an example exercise, intended to identify differences that may occur across study evaluations when using the SciRAP and IRIS tools, rather than an attempt to validate or draw firm conclusions about the use of any one particular tool or method for conducting study evaluations in a broader context.

It might be expected that study evaluations conducted by untrained reviewers would result in greater variation between evaluations and between tools compared to more experienced reviewers. However, this was not apparent in this case study, and final conclusions about study confidence did not vary greatly between the reviewers. As this is only an example exercise however, it is possible that in other circumstances the impact of training with the tools, as well as level of experience, on study evaluation would be more apparent. Furthermore, the results obtained are impacted by the heterogeneity of the included study types. For example, as listed in 5.1; four rat, one hen, two fish and two mouse studies were evaluated. Although this may be a realistic situation for a chemical risk assessment, the range of species, the varying life stage of included animals, the inclusion of in utero as well as adult exposures and the diversity of the nature of the toxic endpoints assessed, are all barriers to identification of differences in evaluated outcomes attributable to the tool being used. Whilst these study evaluations successfully highlight some areas where SciRAP and IRIS differ, this is by no means exhaustive.

In this case study, where differences in evaluations were detected between the two reviewers, no attempts were made to find resolution. In fact, reporting consensus results where there had originally been a discrepancy between evaluators was considered to risk masking the differences the case study was designed to detect. In any case this would ordinarily be a subsequent step in the study evaluation process. SciRAP does not stipulate a method for resolving discrepancies between evaluators, however in practice, the method would be the same for both the IRIS and SciRAP approaches, i.e. by discussion between the reviewers and involving a third reviewer in cases where an agreement cannot be reached. In our experience, reaching a consensus across reviewers using the SciRAP tool is greatly facilitated by being able to compare reviewers’ judgments of the detailed criteria (Beronius and Ågerstrand 2017).

### 5.3. Main differences between the SciRAP and IRIS tools and potential impact on evaluation

In many cases SciRAP criteria were found to closely match the components listed in the guidance for the IRIS tool. For example, the IRIS prompting question “Was the duration and frequency of exposure sensitive for detecting the outcome of interest?” was matched by SciRAP criteria “The timing and duration of administration were appropriate for investigating the included endpoints” and “The frequency of administration was appropriate for investigating the included endpoints”. In some instances, SciRAP criteria added more detail, for example addressing further subdivisions of components referring to ‘reporting’, ‘conflict of interests’, ‘experimental conditions’, ‘timing duration and frequency of exposure’ and ‘sensitivity of the procedures for evaluating the outcome of interest’. Consequently, it was easy to identify where SciRAP criteria did and did not corresponded to an IRIS domain.

SciRAP does not include a criterion for addressing attrition. In the case study, the grading for this domain using the SciRAP tool was based only on the level of reporting. Four of seven instances where evaluation gradings differed substantially between tools and reviewers occurred in the domain “Selective Reporting and Attrition”. Consequently, it can be argued that SciRAP is less sensitive to detecting deficiencies in this domain in animal studies, and that inclusion of additional criteria especially targeting attrition may be a focus of future development of the SciRAP tool.

Similarly, SciRAP was found not to address the domain for observational bias/blinding. In general terms, lack of blinding during outcome assessment has been found to result in a tendency to exaggerate treatment effects in animal studies (Bello et al. 2014). However, it has also been argued that blinding in animal studies is not always practical or feasible (Morris 2012) and in some cases it is unethical to do so. This contention is reflected in the guidance provided in relation to consideration of blinding in SciRAP criterion 18. The potential impact of lack of blinding is dependent on the endpoint being evaluated, e.g. blinding of personnel during outcome assessment is more critical if behavioral endpoints are being scored by observation than if hematological endpoints are being automatically analyzed. Equally, it is an important tenet of good laboratory animal care to be able to identify individuals in the case they become unwell or injured. This is not only an ethical argument but may additionally impact the results obtained. Therefore, the true impact of SciRAP less readily detecting observational bias by not addressing blinding is dependent on the study or endpoint being evaluated.

In the case study, the IRIS evaluation for six out of the nine studies resulted in that blinding/allocation concealment was judged as ‘not reported’ by both reviewers. The IRIS tool goes on to categorize the interpreted impact of this lack of reporting on overall confidence in the study results based on the type of outcome (e.g., less concern is raised regarding lack of blinding for highly automated methods). In three of the cases the lack of reporting was judged to not impact overall quality significantly, and the domain was judged as “adequate” (NR+) by both reviewers. However, in the remaining three cases, both reviewers concluded that lack of reporting raised important concerns, resulting in the judgment “deficient” (NR-).

The SciRAP tool also lacks specific criteria for evaluation of the IRIS domain, “Results presentation”. It is difficult to judge how this would impact the final grading. Generally, in our experience, this is less often found deficient during evaluations with the IRIS tool, but when limitations are flagged here, it is typically very impactful. Such limitations include, for example, a study reporting a continuous outcome but not reporting variability, developmental data being presented on a pup rather than a litter basis, or pooling of data for both sexes when outcomes are known to have sex-specific differences in response. In contrast to the IRIS tool, as well as the ToxRTool and OHAT tool, the SciRAP tool does not provide a predefined method for categorizing the final outcome of the study evaluation, for example into reliability categories or with final overall grading of domains or the study as a whole. The colour profile and score generated by the SciRAP online tool is intended to be interpreted by the evaluator on a case-by-case basis and using a method that fits the purpose of the evaluation and any regulatory requirements. As such, the case presented here is an example of one way in which the output of SciRAP evaluations can be used to draw conclusions. Any principles for grading domains or otherwise categorizing studies based on the output of the SciRAP tool need to be carefully considered in light of the consequences for the final conclusion of study quality. As such, an additional consideration for the discrepancies in evaluation between the tools in the case study is how the principles for translating the SciRAP evaluation into IRIS grading were formulated. Although they were carefully developed to be in line with the considerations for grading in IRIS, it may be that further adjustment of the principles would result in even better concordance in grading between the tools.

Fundamentally, the significance of similarities and differences between SciRAP and IRIS, but also OHAT and ToxRTool on overall study confidence judgements is context dependent. In other words, different elements of internal validity, sensitivity or reliability may be more or less important for study evaluation in different cases, depending on the application of the study evaluation tool, the type of studies or endpoints. There may also be aspects of study quality that are especially challenging to evaluate. One such aspect, in our experience, is the evaluation of the appropriateness of statistical analysis, which may require the specific expertise of statisticians. At the same time, it is important that the biological and toxicological significance of the study is considered within the context of the study evaluation. Overall, the critical point is that the evaluation of individual study quality is structured, detailed enough and sufficiently recorded to enable transparency in this process. The documentation is important in later steps, when comparing outcomes across several studies. For example, it is necessary to be able to determine whether conflicting results between studies may be explained by specific differences in the conduct or design of the study that are reflected in the study quality. A structured and well-reported evaluation of study quality may also be useful for consequent steps of evaluating the human relevance, as well as generalizability, of evidence. Certain aspects of study quality, such as study design issues related to the animal model or dosing regimen, may for example be relevant for the determination of human relevance.

## 6. Considerations regarding evaluation of study sensitivity

Study sensitivity describes the extent to which various study features enable a true effect to be detected, if one is present. This may involve evaluating aspects of study design and conduct, which is why sensitivity can be considered as a component of internal validity (Cooper et al., 2016). In the context of *in vivo* animal toxicology studies, assessing study sensitivity may include evaluation of the chosen animal model, the animal housing conditions, the route and timing of chemical administration and the sensitivity and timing of outcome measurements. Such aspects are not strictly encompassed in a RoB assessment, which comparatively focuses on detection of systematic errors.

Within the field of regulatory health risk assessment, study sensitivity is an essential consideration in determining whether negative results can be relied upon i.e. whether a chemical exposure did or did not cause the effect in question, or simply whether the test system was unable to detect it. More broadly, evaluation of study sensitivity has importance in determining whether a study is adequately able to address the question posited in a given risk assessment; even in cases where the RoB is determined to be low. Furthermore, inconsistent results across a number of studies addressing the same risk assessment question may be better understood following careful examination of study sensitivity and this may help avoid inadvertently drawing false conclusions (Cooper et al., 2016).

Owing to the particular relevance of sensitivity to health risk assessment, SciRAP includes several criteria to specifically target study sensitivity, and similarly, the IRIS tool explicitly addresses study sensitivity under a specific separate domain to RoB. It is not commonly a feature of RoB evaluation tools to incorporate sensitivity analysis (Cooper et al., 2016) although in some instances, such as the OHAT systematic review approach, aspects of study sensitivity are addressed in a later step outside of the RoB evaluation.

Within the IRIS tool, inclusion of a dedicated domain addressing sensitivity places emphasis on evaluating reported study effects as truly detected or being potentially misleading (e.g. false negative results); and distinguishes this evaluation from consideration of RoB as commonly applied in systematic review. The separate domains also assists in creating transparent ties between the strengthens and weakness identified during study evaluation and later stages of systematic review involving evidence synthesis and drawing supported conclusions. Therefore, a strength of the IRIS approach is simultaneous evaluation of traditional RoB domains (to address potential factors that might induce systematic errors) and sensitivity domains (to address potential factors that limit the ability of a study to detect a true effect). Ultimately, evaluation of both RoB and sensitivity is important to identify the most credible results, and to facilitate comparisons of results across sets of studies on a given outcome.

## 7. The role of expert judgement in study evaluation.

A shared aspect of the SciRAP and IRIS tools is in producing targeted expert evaluations in a structured and transparent manner which can be used to support a final conclusion. Expert judgment is an integral and necessary part of study evaluation, as well as other steps of hazard and risk assessment (e.g. SCHEER 2018), and the intention of these tools is not to restrict or replace expert judgment. It should also be noted that both these tools strive for harmonization of study evaluation between reviewers while remaining flexible enough to be applicable to a wide variety of animal *in vivo* studies and accounting for heterogeneity in terms of different study designs and methods. The criteria-based approach guides reviewers to consider the same study details, promoting harmonization of study evaluation. However, the criteria need to be general enough to be applicable for different study types. To further promote harmonization of how the different criteria are judged, the SciRAP tool includes specific guidance for each criterion. However, this does not ensure that different reviewers judge criteria in the same way (Beronius and Ågerstrand, 2017). The domain-based approaches reviewed here consist of fewer but more open-ended questions for the reviewer to consider, striving for a balance between flexibility (i.e., being applicable to a range of heterogeneous study types) and simplicity (i.e. ease of use). To promote harmonization, the IRIS approach provides reviewers with detailed considerations and example answers to establish basic parameters for the evaluation of each domain. Structured incorporation of expert judgment is built into the evaluation process by encouraging reviewers to add specificity, where needed, to their answers. This allows the general IRIS tool to remain flexible and adaptable to a range of chemical- and assessment-specific needs (and encouraging reviewers to carefully review the literature database).

## 8. Conclusions

This paper describes our experience evaluating reliability and risk of bias of animal toxicity studies, using different tools. Overall, considerable but not complete overlap was found when comparing the scope of four study evaluation tools explored here: two domain based tools designed for use in systematic review context (IRIS and OHAT) and two criteria based tools designed for application in regulatory hazard and risk assessment (SciRAP and ToxRTool). The SciRAP tool was found to cover the majority of the elements included for study evaluation in the IRIS tool, which we used as basis for our comparisons, including specific considerations for evaluating study sensitivity and RoB. SciRAP also includes additional elements, such as the appropriateness of statistical methods and disclosure of funding sources and conflicts of interest. However, considerations for blinding, attrition and results presentation, which are important elements to evaluate as part of a RoB assessment, are not specifically addressed in SciRAP. The two domain-based tools, OHAT and IRIS were found to be similar in scope but differed in their approach to consideration of study sensitivity and in the results presentation domain. By comparison ToxRTool met fewer of the IRIS domains, focusing on assessing completeness of reporting.

One strength of the SciRAP tool is that it provides an approach for structured evaluation of reporting quality that is clearly separated from the evaluation of methodological quality. The issue of insufficient reporting, how it hampers evaluation of the reliability of study results, and its implications both for the quality of research and for hazard and risk assessment of chemicals has been widely discussed (e.g. Glasziou et al., 2014; Stark 2018; Ågerstrand et al. 2018). Within systematic review, since study evaluation is very much focused on evaluating internal validity or methodological quality, it is important to separate the evaluation of reporting quality from methodological quality. Still, it is critical that reviewers are able to characterize deficiencies in reporting, since it may explain cases where methodological quality could not be adequately evaluated.

The case study conducted here highlights some aspects of the SciRAP tool that could be adjusted to increase similarity with study quality evaluation in a systematic review context. It should be noted that this study was not intended as a validation of the SciRAP tool but as an exploratory study to provide information for further tool development. In this case, the conclusions regarding overall confidence in the nine evaluated studies based on evaluations using the SciRAP tool did not differ significantly from conclusions based on evaluations in the IRIS tool. Thus, it seems that the lack of specific criteria for considering blinding, attrition and results presentation in the SciRAP tool did not overall impact study evaluation in this case. However, with the movement towards applying systematic review methodologies, including evaluation of RoB, in hazard and risk assessment (EFSA 2010; Rhomberg et al. 2013; Rooney et al. 2014; Whaley et al. 2016; Woodruff and Sutton 2011), it is acknowledged that it may be useful to update the SciRAP tool with additional criteria and guidance specifically covering these study elements. One important intention of SciRAP is to provide tools that are flexible and can be applied for study evaluation in different contexts.

Overall, an important conclusion from this work is that tools that provide a detailed, structured and predefined method for evaluating study quality promote consistency between reviewers and, above all, transparency in study evaluation. These are essential aspects for evaluation and integration of evidence in health risk assessment of chemicals, and a prerequisite for drawing conclusions regarding chemical health risks on which regulatory decision making is based.

## Supplementary Material

Supplement1

## Figures and Tables

**Fig. 2a.**
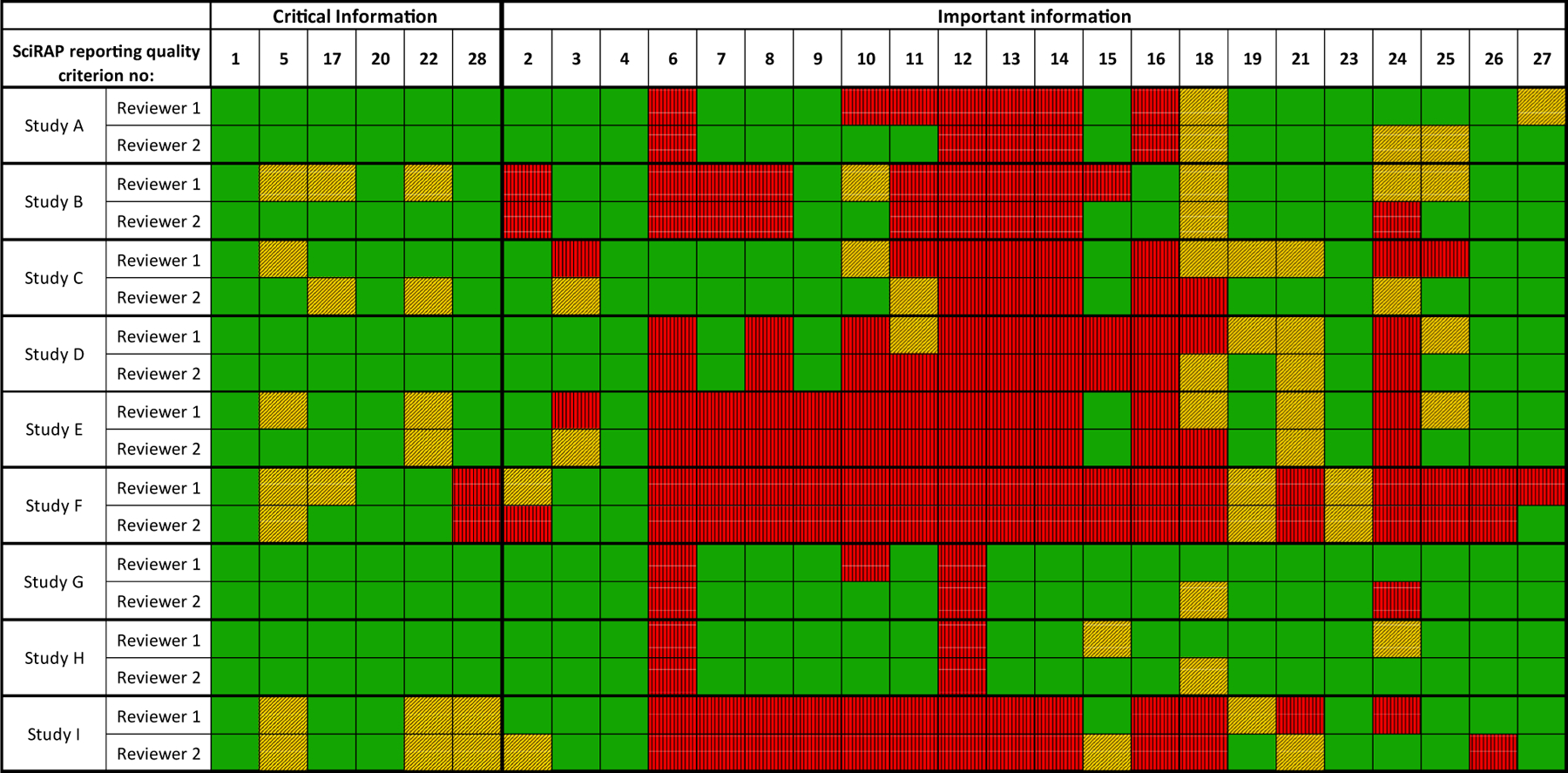
SciRAP evaluations of the reporting quality of nine *in vivo* studies evaluating developmental toxicity of TPhP organized according to the IRIS approach for evaluating the Reporting domain. Green cells indicate criteria that were judged as “fulfilled”, yellow cells with diagonal bars indicate criteria that were judged as “partially fulfilled”, red cells with vertical bars indicate criteria that were judged as “not fulfilled”. The criteria are listed and numbered in Supplemental Table 2.

**Fig. 2b.**
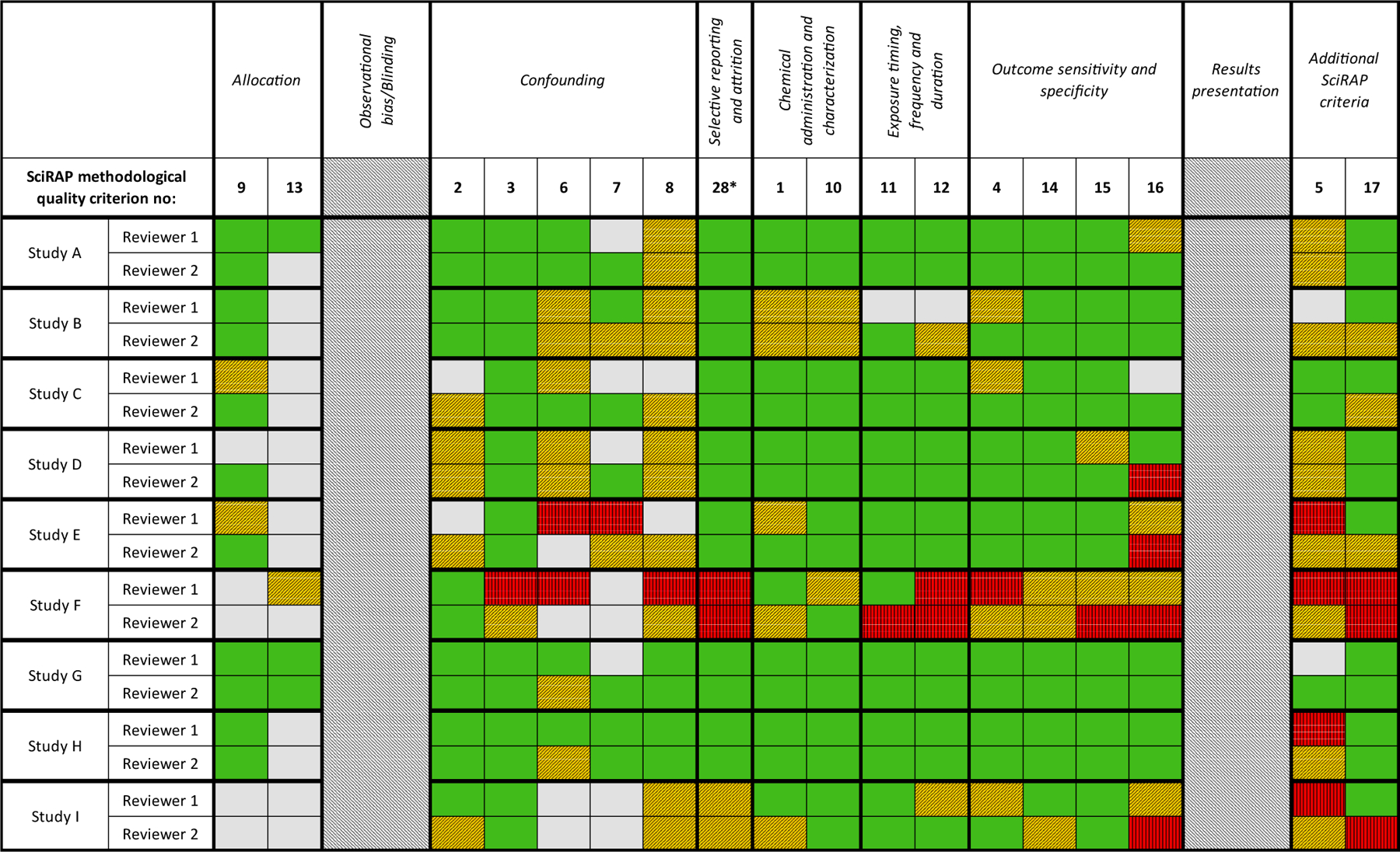
SciRAP evaluations of the methodological quality of nine *in vivo* studies evaluating developmental toxicity of TPhP organized according to the structure of the IRIS tool. Green cells indicate criteria that were judged as “fulfilled”, yellow cells with diagonal bars indicate criteria that were judged as “partially fulfilled”, red cells with vertical bars indicate criteria that were judged as “not fulfilled, grey cells indicate criteria that were judged as “not determined”. Dashed columns indicate that no SciRAP criteria match this specific IRIS subdomain. The criteria are listed and numbered in Supplemental Table 3. * The domain “Selective reporting or attrition” was judged to only be covered by the SciRAP reporting quality criterion 28.

**Table 1:** Evaluation categories and domains in the IRIS *in vivo* animal tool. The nine domains were used to create a framework for comparisons between the ToxRTool, SciRAP, OHAT and IRIS tools.

Evaluation Categories	Evaluation Domains
Reporting Quality	1. Reporting
Risk of Bias	2. Allocation 3. Observational Bias/Blinding 4. Confounding 5. Selective Reporting and Attrition
Study Sensitivity	6. Chemical Administration and Characterization 7. Exposure Timing, Frequency, and Duration 8. Outcome Sensitivity and Specificity 9. Results Presentation

**Table 2:** Comparison of the coverage of different elements of study evaluation among the ToxRTool, SciRAP, OHAT and IRIS tools.

	IRIS	OHAT	SciRAP^1^	ToxRTool
**Reporting**	Prompting questions: – Critical information necessary for study evaluation: Species; test article name; levels and duration of exposure; route; qualitative or quantitative results for at least one outcome of interest. – Important information necessary for study evaluation: aspects related to the test animal, exposure methods, experimental design, and outcome evaluation methods.	Reporting is **partially addressed.** *Reporting only addressed in the OHAT data extraction tool.*	Reporting is **addressed** by the SciRAP reporting quality criteria.	Reporting is **partially addressed** but is not distinguished from criteria addressing methodological quality or other IRIS domains.
**Allocation**	Prompting questions: – Did each animal or litter have an equal chance of being assigned to any experimental group (i.e., random allocation)? – Is the allocation method described? – Aside from randomization, were any steps taken to balance variables across experimental groups during allocation?	**Addressed** by RoB question: “Was administered dose or exposure level adequately randomized?”	**Addressed** by two methodological quality criteria.	*Randomization* ***not addressed***
**Observational bias/blinding**	Prompting questions: – Does the study report blinding or other methods/procedures for reducing observational bias? – If not, did the study use a design or approach for which such procedures can be inferred? – What is the expected impact of failure to implement (or report implementation) of these methods/procedures on results?	**Addressed** by RoB question: “Was allocation to study groups adequately concealed?”	*Blinding/Allocation concealment* ***not addressed***.	*Blinding/Allocation concealment* ***not addressed***.
**Confounding**	Prompting questions: – Are there differences across the treatment groups (e.g., co-exposures, vehicle, diet, palatability, husbandry, health status, etc.) that could bias the results? – If differences are identified, to what extent are they expected to impact the results?	**Addressed** by RoB question: “Were experimental conditions identical across study groups?”	**Addressed** by five methodological quality criteria.	*Confounding* ***not addressed***.
Selective reporting and attrition	Prompting questions: – Are all results presented for outcomes described in the methods? – Are all animals accounted for in the results? If there are discrepancies, do authors provide an explanation?	**Addressed** by RoB questions: “Were all measured outcomes reported?” and “Were outcome data complete without attrition or exclusion from analysis?”	Selective reporting **partially addressed** by reporting quality criteria.*Attrition not addressed.*	Selective reporting **partially addressed**.*Attrition not addressed.*
**Chemical administration and characterization**	Prompting questions: – Does the study report the source and purity and/or composition of the chemical? If not, can the purity and/or composition be obtained from the supplier? – Was independent analytical verification of the test article purity and composition performed? – Did the authors take steps to ensure the reported exposure levels were accurate? – Are there concerns about the methods used to administer the chemical?	**Addressed** by RoB question: “Can we be confident in the exposure characterization?”	**Addressed** by two methodological quality criteria.	**Addressed** by three criteria.
**Exposure timing, frequency** and duration	Prompting questions: – Does the exposure period include the critical window of sensitivity? – Was the duration and frequency of exposure sensitive for detecting the outcome of interest?	Exposure timing, frequency and duration is “**partially addressed**”.*Only addressed in the OHAT approach consideration of indirectness.*	**Addressed** by two methodological quality criteria.	**Addressed** by one criterion.
**Outcome sensitivity and specificity**	Prompting questions: – Are there concerns regarding the timing of the outcome assessment? – Are there concerns regarding the sensitivity and specificity of the protocols? – Are there concerns regarding the validity of the protocols? – Are there serious concerns regarding the sample size?	**Partially addressed** by RoB question: “Can we be confident in the outcome assessment?”*Validity of the protocol and adequacy of the sampling not addressed.*	**Partially addressed** by four methodological quality criteria.*Validity of the protocol not addressed*	**Partially addressed** by one criterion.*Validity of the protocol not addressedAdequate sampling not addressed*
**Results presentation**	Prompting questions: – Does the level of detail allow for an informed interpretation of the results? – Are the data analyzed, compared, or presented in a way that is inappropriate or misleading?	*Presentation of results* ***not addressed***.	*Presentation of results* ***not addressed***.	**Partially addressed** by one criterion.
**Additional aspects**	–	Specifically addresses the inclusion of a concurrent negative control.Addresses the appropriateness of the statistical methods used in the study.Includes RoB domain for “other” study elements that can have an impact on study validity or reliability, for example “unintended co-exposures”.	Specifically addresses the inclusion of a concurrent negative control.Addresses the appropriateness of the statistical methods used in the study.Addresses disclosure of funding sources and conflicts of interest.Addresses individual identification of animals.Includes a criterion for “other” study elements that can have an impact on study validity or reliability, for example “blinding”.	Specifically addresses the inclusion of a concurrent negative control.Addresses the appropriateness of the statistical methods used in the study.

1 For information on which SciRAP criteria were considered to cover the different IRIS domains, please see Figs. 2a and 2b.

**Table 3:** Principles for translating SciRAP reporting quality criteria into IRIS rating for the domain “Reporting quality”.

IRIS rating	Principles
Good (++)	All SciRAP reporting criteria corresponding to “critical information”^1^ listed in the IRIS approach were evaluated as “fulfilled”. All SciRAP reporting criteria corresponding to “important information”^2^ listed in the IRIS approach were evaluated as “fulfilled” or “partially fulfilled”.
Adequate (+)	All SciRAP reporting criteria corresponding to “critical information” listed in the IRIS approach were evaluated as “fulfilled” or “partially fulfilled”. Some SciRAP reporting criteria corresponding to “important information” listed in the IRIS approach were evaluated as “not fulfilled”. However, the missing information is not expected to significantly impact the study interpretation.
Deficient (−)	All SciRAP reporting criteria corresponding to “critical information” listed in the IRIS approach were evaluated as “fulfilled” or “partially fulfilled”. Some SciRAP reporting criteria corresponding to “important information” listed in the IRIS approach were evaluated as “not fulfilled”, and the missing information is expected to significantly reduce the ability to evaluate the study.
Critically deficient (−)	One or more of the SciRAP reporting criteria corresponding to “critical information” listed in the IRIS approach were evaluated as “not fulfilled”.

1 Critical information necessary to perform study evaluation according to the IRIS approach: species; test article name; levels and duration of exposure; route (e.g., oral; inhalation); qualitative or quantitative results for at least one outcome of interest.

2 Important information for evaluating the study methods according to the IRIS approach: Test animal strain, sex, source, and general husbandry procedures; Exposure methods, i.e. source, purity, method of administration; Experimental design, i.e. frequency of exposure, animal age and life stage during exposure and at outcome evaluation; Outcome evaluation methods, i.e. assays or procedures used to measure the outcomes of interest.

**Table 4:** Principles for translating SciRAP methodological quality criteria into IRIS rating for the domains “Selection and performance bias”, “Confounding/variable Control”, “Reporting and Attrition bias”, “Exposure methods sensitivity” and “Outcome measures and results display”.

IRIS rating	Principles
Good (++)	All SciRAP criteria matched to this domain were evaluated as “fulfilled”.
Adequate (+)	All SciRAP criteria matched to this domain were evaluated as “fulfilled” or “partially fulfilled”. Single criteria may be evaluated as “not fulfilled” or “not determined” if not judged to significantly influence the rating of the domain.
Deficient (−)	Several of the SciRAP criteria matched to this domain were judged as “not fulfilled”.
Critically deficient (−)	All the SciRAP criteria matched to this domain were evaluated as “not fulfilled”.
Not reported but interpreted as adequate (NR+)	All SciRAP criteria matched to this domain were evaluated as “not determined” based on insufficient reporting. However, it is not likely that any unreported information may have substantially impacted the results or biased the results towards the null.
Not reported and interpreted as deficient (NR−)	All SciRAP criteria matched to this domain were evaluated as “not determined” based on insufficient reporting, and it is likely that the unreported information may have substantially impacted the results or biased the results towards the null.^1^

1 For the domain “Allocation”, if information regarding the allocation to treatment groups is missing this should always be interpreted as deficient (NR−).

## Text Box 1 Different terminology used to describe study quality.

**Table:**

Term.	Definition.	References.
**Reliability.**	“The inherent quality of a test report or publication relating to preferably standardized methodology and the way the experimental procedure and results are described to give evidence of the clarity and plausibility of the findings”	ECHA, 2011; Klimisch et al. 1997; OECD, 2005
**Internal Validity**	“Measure of the credibility of study findings that reflects the ability of a study's design and conduct to protect against systematic errors that may bias (over- or under-estimate) the results or estimate of effect”	Rooney et al., 2016
**Risk of Bias**	The risk for systematic errors (bias) that can lead to underestimation or overestimation of the true effect in a study.	Higgins and Green 2011
**Study Quality**	“A complex idea with different meanings for different groups including one or more of the following: reporting quality, applicability and risk of bias.”	Rooney et al., 2016
**Methodological Quality**	How extensively systematic errors (bias) are likely to have been prevented as a result of the design and conduct of the study, resulting in detection of “true” findings.	Olivo et al., 2008, Samuel et al. 2016
**Reporting Quality**	The extent to which a complete and transparent description of the study design, conduct and analysis is provided.	Moher et al., 1995
