# Supplementary material for: Evaluating reliability and risk of bias of in vivo animal data for risk assessment of chemicals – Exploring the use of the SciRAP tool in a systematic review context
Source: Environ Int. Author manuscript; Available in PMC 2024 Jul 9. (PMC11231916; doi:10.1016/j.envint.2020.106103)
Supplement: Supplement1 [file NIHMS2000285-supplement-Supplement1.docx]

**Supplemental Material**

**Supplemental table 1.** ToxRTool criteria for assessing reliability quality of *in vivo* toxicity studies. <https://ec.europa.eu/jrc/en/eurl/ecvam> Accessed January 2018. Criteria indicated in red have to be fulfilled in order for the study to be categorised as Klimish reliability category 1 (reliable without restriction) or 2 (reliable with restrictions).

| **Criteria Group I: Test substance identification** |
| --- |
| 1. Was the test substance identified? |
| 1. Is the purity of the substance given? |
| 1. Is information on the source/origin of the substance given? |
| 1. Is all information on the nature and/or physico-chemical properties of the test item given, which you deem indispensable for judging the data (see explanation for examples)? |
| **Criteria Group II: Test organism characterisation** |
| 1. Is the species given? |
| 1. Is the sex of the test organism given? |
| 1. Is information given on the strain of test animals plus, if considered necessary to judge the study, other specifications (see explanation for examples)? |
| 1. Is age or body weight of the test organisms at the start of the study given? |
| 1. For repeated dose toxicity studies only (give point for other study types): Is information given on the housing or feeding conditions? |
| **Criteria Group III: Study design description** |
| 1. Is the administration route given? |
| 1. Are doses administered or concentrations in application media given? |
| 1. Are frequency and duration of exposure as well as time-points of observations explained? |
| 1. Were negative (where required) and positive controls (where required) included (give point also, when absent but not required, see explanations for study types and their respective requirements on controls)? |
| 1. Is the number of animals (in case of experimental human studies: number of test persons) per group given? |
| 1. Are sufficient details of the administration scheme given to judge the study (see explanation for examples)? |
| 1. For inhalation studies and repeated dose toxicity studies only (give point for other study types): Were achieved concentrations analytically verified or was stability of the test substance otherwise ensured or made plausible? |
| **Criteria Group IV: Study results documentation** |
| 1. Are the study endpoint(s) and their method(s) of determination clearly described? |
| 1. Is the description of the study results for all endpoints investigated transparent and complete? |
| 1. Are the statistical methods applied for data analysis given and applied in a transparent manner (give also point, if not necessary/applicable, see explanations)? |
| **Criteria Group V: Plausibility of study design and results** |
| 1. Is the study design chosen appropriate for obtaining the substance-specific data aimed at (see explanations for details)? |
| 1. Are the quantitative study results reliable (see explanations for arguments)? |

**Supplemental Table 2.** SciRAP criteria for assessing reporting quality of *in vivo* toxicity studies. Version 19 January, 2018. Available online at www.scirap.org.

| **Test compound and controls** |
| --- |
| 1. The chemical name, ID or CAS-number of the test compound was given. |
| 1. The purity of the test compound was stated or is traceable according to information given regarding manufacturer and lot/batch number. In case of mixtures, the composition of different constituents was stated. |
| 1. The vehicle was described. |
| 1. It was stated that a negative control group was included. |
| **Animal model and housing conditions** |
| 1. The animal model (species, strain, age or life stage and sex) was described. |
| 1. The method for individual identification of animals was described. |
| 1. The housing temperature was stated. |
| 1. The relative humidity was stated. |
| 1. The light-dark cycle was described. |
| 1. The number of animals per sex in each cage was stated. |
| 1. The cage materials were described. |
| 1. Any materials used for physical enrichment were described. |
| 1. Water bottle materials were described. |
| 1. The bedding material used was described. |
| 1. The type and source of feed were reported. |
| 1. The source of drinking water was reported. |
| **Dosing and administration of the test compound** |
| 1. The administered dose levels or concentrations were stated. |
| 1. The method for allocating animals to different treatments was stated. |
| 1. The total number of animals per dose group was stated. |
| 1. The route of administration was stated. |
| 1. The sex and age (or life stage) of the animals at start of dosing was stated or is obvious from the information given, e.g. “pregnant rats were used” is enough information that animals are female and sexually mature/adult. |
| 1. The frequency and duration of dosing/administration of the test compound was stated. |
| **Data collection and analysis** |
| 1. The test and/or analytical methods used were sufficiently described to allow for evaluation of the reliability of results. |
| 1. The method for allocating animals to different tests and measurements (e.g. tissue collection or evaluation of functional or behavioural endpoints) was stated. |
| 1. The sex, age and number of animals per dose group subjected to separate tests and measurements was stated. |
| 1. The statistical methods and software used were described. |
| 1. The statistical unit, e.g. the individual or the litter, was stated. |
| 1. All results for the investigated endpoints were reported. The most critical results were presented in tables and figures, including description of variation and statistically significant results. |
| **Funding and competing interests** |
| 1. The funding sources for the study were stated. |
| 1. Any competing interests were disclosed or it was explicitly stated that the authors did not have any competing interests. |

**Supplemental Table 3.** SciRAP criteria for assessing methodological quality of *in vivo* toxicity studies. Version 19 January 2018. Available online at [www.scirap.org](http://www.scirap.org).

| **Test compound and controls** |
| --- |
| 1. The test compound or mixture was unlikely to contain any impurities that may significantly have affected its toxicity. |
| 1. An appropriate vehicle was used that is not expected to interfere with the absorption, distribution, metabolism, excretion or toxicity of the test compound. |
| 1. A concurrent negative control group was included. |
| **Animal model and housing conditions** |
| 1. A reliable and sensitive animal model was used for investigating the test compound and selected endpoints. |
| 1. Animals were individually identified. |
| 1. Housing conditions (temperature, relative humidity, light-dark cycle) were appropriate for the study type and animal model. |
| 1. The number of animals per sex in each cage were appropriate for the study type and animal model. |
| 1. The test system is unlikely to contain contaminants that could affect study results, such as organic pollutants, pesticide residues, heavy metals, and mycotoxins, as well as phytoestrogens. |
| **Dosing and administration of test compound** |
| 1. The allocation of animals to different treatments was randomized. |
| 1. The route of administration was appropriate and not likely to interfere with the study results. |
| 1. The timing and duration of administration were appropriate for investigating the included endpoints. |
| 1. The frequency of administration is appropriate for investigating the included endpoints. |
| **Data collection and analysis** |
| 1. The allocation of animals to different tests and measurements was randomized. |
| 1. Reliable, and sensitive test methods were used for investigating the selected endpoints. |
| 1. Measurements were collected at suitable time points in order to generate sensitive, valid and reliable data. |
| 1. A sufficient number of animals per dose group were subjected to separate tests/data collection/measurements to generate reliable and valid results. |
| 1. The statistical methods have been clearly described and do not seem inappropriate, unusual or unfamiliar. |
|  |
| 1. Are there any other aspects of study design, performance or reporting that influence reliability? |

**Supplemental table 4.** List of RoB domains and questions from “OHAT Risk of Bias Rating Tool for Human and Animal Studies”. Available online at <https://ntp.niehs.nih.gov/pubhealth/hat/review/index-2.html>. Accessed March, 2018.

| **Selection bias** |
| --- |
| 1. Was administered dose or exposure level adequately randomized? |
| 1. Was allocation to study groups adequately concealed? |
| 1. Did selection of study participants result in appropriate comparison groups? |
| **Confounding Bias** |
| 1. Did the study design or analysis account for important confounding and modifying variables? |
| **Performance Bias** |
| 1. Were experimental conditions identical across study groups? |
| 1. Were the research personnel and human subjects blinded to the study group during the study? |
| **Attrition/Exclusion Bias** |
| 1. Were outcome data complete without attrition or exclusion from analysis? |
| **Detection Bias** |
| 1. Can we be confident in the exposure characterization? |
| 1. Can we be confident in the outcome assessment? |
| **Selective Reporting Bias** |
| 1. Were all measured outcomes reported? |
| **Other Bias** |
| 1. Were there no other potential threats to internal validity (e.g., statistical methods were appropriate and researchers adhered to the study protocol)? |

**Supplemental Table 5.** Details of the nine in vivo studies included in the case study.

| Study ID | Study Overview | Species | Reference |
| --- | --- | --- | --- |
| A | Evaluation of the metabolic profiles of UCD-T2DM rats exposed to dietary Triphenyl phosphate from gestational day 8.5 to weaning. Animals were assessed from 3.5 to 6 months of age for development of diabetes and obesity. | UCD-T2DM rat | Green AJ, Graham JL, Gonzalez EA, et al. 2017. Perinatal triphenyl phosphate exposure accelerates type 2 diabetes onset and increases adipose accumulation in UCD-type 2 diabetes mellitus rats. Reproductive Toxicology. 68:119-129 |
| B | Study investigating the metabolic and developmental effects of C57Bl/6 mice exposed to Triphenyl phosphate during gestation. | C57Bl/6 mouse | Philbrook NA, Restivo VE, Belanger CL, Winn LM. 2018. Gestational triphenyl phosphate exposure in C57Bl/6 mice perturbs expression of insulin-like growth factor signaling genes in maternal and fetal liver. Birth Defects Research. 1-12 |
| C | Study investigating the reproductive and teratogenic effects of Triphenyl phosphate on Sprague Dawley rats exposed in a 91-day sub-chronic feeding study. | Sprague Dawley rat | Welsh JJ, Collins TF, Whitby KE. 1987. Teratogenic potential of triphenyl phosphate in Sprague-Dawley rats. |
| D | Investigation of the metabolic effects of Triphenyl phosphate and diphenyl phosphate on neonatal ICR mice. Includes evaluation of exposure on reproductive organ development and endocrine systems, glucose tolerance and effect on metabolomic profile. | ICR mouse | Wang D, Zhu W, Chen L, et al. 2018. Neonatal triphenyl phosphate and its metabolite diphenyl phosphate exposure induce sex- and dose-dependent metabolic disruptions in adult mice. Environmental Pollution. 237:10-17 |
| E | Assessment of immunotoxicity of Sprague-Dawley rats exposed to Triphenyl Phosphate in a sub-chronic feeding study for 120 days from weaning. | Sprague Dawley rat | Hinton DM, Jessop JJ, Arnold A et al. 1987. Evaluation of immunotoxicity in a subchronic feeding study of triphenyl phosphate. Toxicology and Industrial Health. 3(1):71-89 |
| F | Evaluation of acute dermal and oral toxicity of phosphate esters in adult Sprague-Dawley rats and White Leghorn hens. An extended oral dosing regimen lasting 42 days was used to assess neurotoxicity in hens. | White Leghorn hen | Johannsen FR, Wright PL, Gordon DE, et al. 1977. Evaluation of Delayed Neurotoxicity and Dose-Response Relationships of Phosphate Esters in the Adult Hen. Toxicology and Applied Pharmacology. 41:291-304 |
| G | Investigation of the effects of Triphenyl phosphate on thyroid hormone regulation and its genetic control in zebrafish embryos. | Zebrafish | Kim S, Jung J, Lee I, et al. 2015. Thyroid disruption by triphenyl phosphate, an organophosphate flame retardant, in zebrafish embryos/larvae, and in GH3 and FRTL-5 cell lines. Aquatic Toxicology. 160:188-196 |
| H | Study examining the effects of exposure to Triphenyl phosphate on zebrafish larvae/embryos exposed from 4 to 120 hpf. Primarily focuses on endocrine disruption and the impact of exposure on survival, hatching, malformation and transcriptomics. | Zebrafish | Liu C, Wang Q, Liang K, et al. 2013. Effects of tris(1,3-dichloro-2-propyl) phosphate and triphenyl phosphate on receptor-associated mRNA expression in zebrafish embryos/larvae. Aquatic Toxicology. 128-129:147-157 |
| I | Study examining the acute toxicity of Triphenyl phosphate when administered by oral and subcutaneous injection to mice, rats and cats. Also, 35-day feeding study examining toxicity of Triphenyl phosphate in rats. | Rat | Sutton WL, Terhaar CJ, Miller FA, et al. Studies on the Industrial Hygiene and Toxicology of Triphenyl Phosphate. Archives of Environmental Health: An International Journal. 1:1, 33-46 |
